# Non-invasive cardiovascular magnetic resonance assessment of pressure recovery distance after aortic valve stenosis

**DOI:** 10.1186/s12968-023-00914-3

**Published:** 2023-01-30

**Authors:** Joao Filipe Fernandes, Harminder Gill, Amanda Nio, Alessandro Faraci, Valeria Galli, David Marlevi, Malenka Bissell, Hojin Ha, Ronak Rajani, Peter Mortier, Saul G. Myerson, Petter Dyverfeldt, Tino Ebbers, David A. Nordsletten, Pablo Lamata

**Affiliations:** 1 School of Biomedical Engineering and Imaging Sciences, King’s College London, London, UK; 2 FEops NV, Ghent, Belgium; 3 Department of Molecular Medicine and Surgery, Karolinska Institutet, Solna, Sweden; 4 Institute for Medical Engineering and Science, Massachusetts Institute of Technology, Cambridge, MA USA; 5 Department of Biomedical Imaging Science, Leeds Institute of Cardiovascular and Metabolic Medicine, University of Leeds, Leeds, UK; 6 Department of Mechanical and Biomedical Engineering, Kangwon National University, Chuncheon, Korea; 7 Cardiovascular Directorate, Guy’s and St Thomas’ NHS Foundation Trust, London, UK; 8 Division of Cardiovascular Medicine, Radcliffe Department of Medicine, Oxford Centre for Clinical Magnetic Resonance Research, University of Oxford, Oxford, UK; 9 Department of Health, Medicine and Caring Sciences, Linköping University, Linköping, Sweden; 10 Center for Medical Image Science and Visualization (CMIV), Linköping University, Linköping, Sweden; 11 Department of Biomedical Engineering and Cardiac Surgery, University of Michigan, Ann Arbor, MI USA

**Keywords:** Aortic stenosis, Pressure recovery, Non-invasive pressure drop, Turbulence, 4D Flow MRI, Flow momentum

## Abstract

**Background:**

Decisions in the management of aortic stenosis are based on the peak pressure drop, captured by Doppler echocardiography, whereas gold standard catheterization measurements assess the net pressure drop but are limited by associated risks. The relationship between these two measurements, peak and net pressure drop, is dictated by the pressure recovery along the ascending aorta which is mainly caused by turbulence energy dissipation. Currently, pressure recovery is considered to occur within the first 40–50 mm distally from the aortic valve, albeit there is inconsistency across interventionist centers on where/how to position the catheter to capture the net pressure drop.

**Methods:**

We developed a non-invasive method to assess the pressure recovery distance based on blood flow momentum via 4D Flow cardiovascular magnetic resonance (CMR). Multi-center acquisitions included physical flow phantoms with different stenotic valve configurations to validate this method, first against reference measurements and then against turbulent energy dissipation (respectively n = 8 and n = 28 acquisitions) and to investigate the relationship between peak and net pressure drops. Finally, we explored the potential errors of cardiac catheterisation pressure recordings as a result of neglecting the pressure recovery distance in a clinical bicuspid aortic valve (BAV) cohort of n = 32 patients.

**Results:**

In-vitro assessment of pressure recovery distance based on flow momentum achieved an average error of 1.8 ± 8.4 mm when compared to reference pressure sensors in the first phantom workbench. The momentum pressure recovery distance and the turbulent energy dissipation distance showed no statistical difference (mean difference of 2.8 ± 5.4 mm, R^2^ = 0.93) in the second phantom workbench. A linear correlation was observed between peak and net pressure drops, however, with strong dependences on the valvular morphology. Finally, in the BAV cohort the pressure recovery distance was 78.8 ± 34.3 mm from *vena contracta*, which is significantly longer than currently accepted in clinical practise (40–50 mm), and 37.5% of patients displayed a pressure recovery distance beyond the end of the ascending aorta.

**Conclusion:**

The non-invasive assessment of the distance to pressure recovery is possible by tracking momentum via 4D Flow CMR. Recovery is not always complete at the ascending aorta, and catheterised recordings will overestimate the net pressure drop in those situations. There is a need to re-evaluate the methods that characterise the haemodynamic burden caused by aortic stenosis as currently clinically accepted pressure recovery distance is an underestimation.

**Supplementary Information:**

The online version contains supplementary material available at 10.1186/s12968-023-00914-3.

## Background

Aortic stenosis (AS) is the most common valvular disease in developed countries, affecting up to 7% of the elderly population. As part of AS risk stratification, clinical guidelines recommend the measurement of the trans-stenotic pressure drop (a more accurate term than the pressure *gradient* used in medical literature) caused by the narrowed aortic valve [1]. In clinical practice this is obtained by Doppler echocardiography and the simplified Bernoulli (SB) equation that provides the *peak* instantaneous pressure drop across the aortic valve. This represents a useful surrogate measure of the haemodynamic burden caused by AS.

**Fig. 1:**
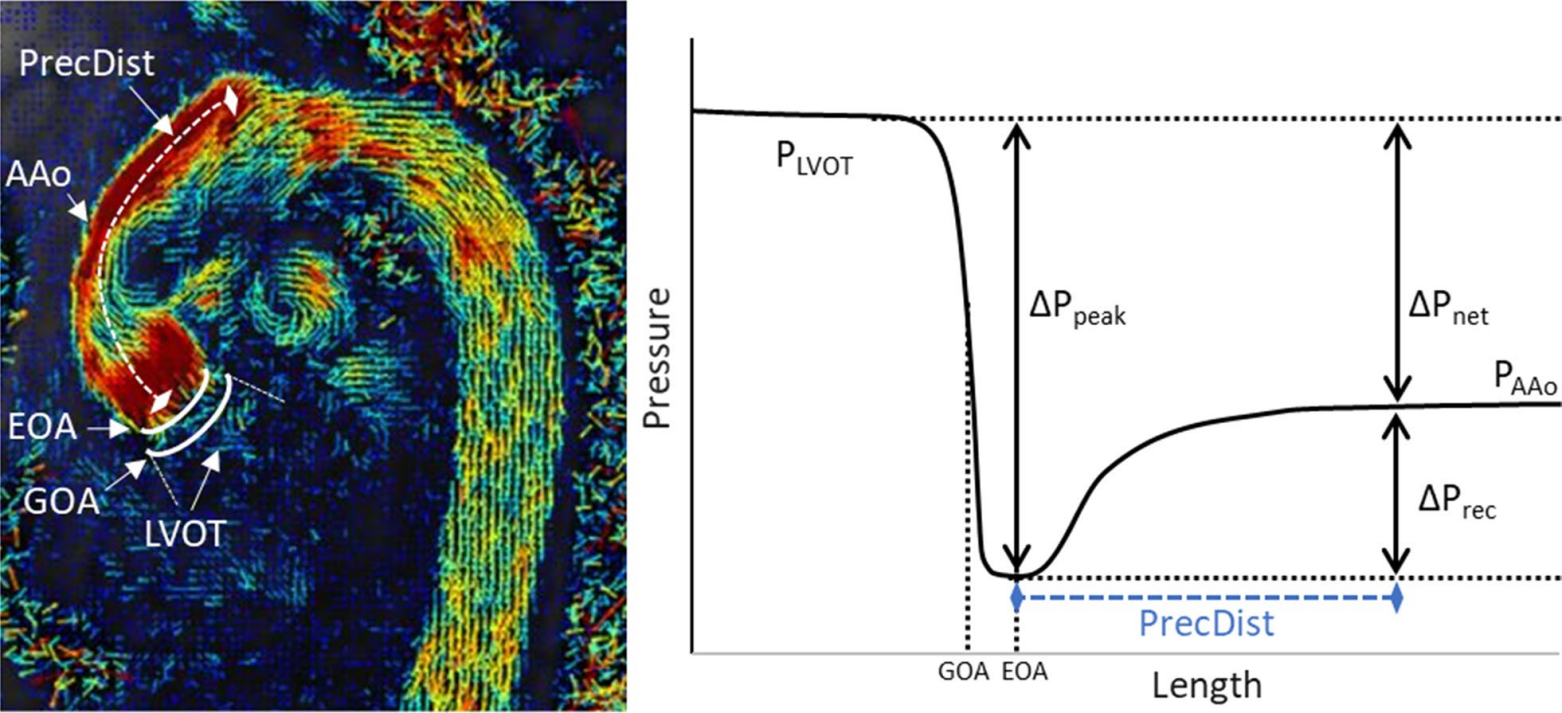
Definition of the pressure concepts in the context of aortic stenosis. Blood flows from left ventricular outflow tract (LVOT) into the ascending aorta (AAo) across the aortic valve that defines the geometric orifice area (GOA). When crossing the valve, a portion of the potential energy of the fluid is converted into kinetic energy, which is maximal at the effective orifice area (EOA) defining the peak pressure drop (ΔP_peak_) where the ejection jet is narrowest. Downstream along the AAo the kinetic energy is transformed back to potential energy until a fully developed laminar flow profile where is the correct point to measure the net pressure drop (ΔP_net_). The pressure recovery (ΔP_rec_) is the difference between ΔP_peak_ and ΔP_net_. The pressure recovery distance (*PRecDist*) is defined as the distance necessary for the blood flow to go from the ΔP_peak_ to ΔP_net_

Invasive cardiac catheterisation may also be used to evaluate the haemodynamic burden of AS by providing the *net* pressure drop across the aortic valve. This however is limited by its invasive nature and inherent risks [2]. The discrepancy between these two measurements of AS severity (peak and net pressure drop) is largely explained by the phenomenon of pressure recovery along the ascending aorta (AAo) as illustrated in Fig. 1 [3, 4], where pressure increases downstream from the AS as a result of the reconversion of kinetic to potential energy. The magnitude of pressure recovery is largely dependent upon the geometry of the aortic valve and the size of the AAo [3, 5].

Non-invasive monitoring of AS severity can potentially be improved by revisiting current simplifications (e.g. SB) towards a more precise and accurate surrogate of the actual burden caused by AS, i.e. the net pressure drop [6]. The effective loss index (ELI) demonstrated the importance of accounting for the geometry of the AAo (i.e., the ratio of diameters) [7, 8]. Other clinical studies have reported that jet eccentricity, effective orifice area (EOA), velocity and ascending aorta diameters are correlated with the pressure recovery [3, 9, 10]. Furthermore, with the development of cardiovascular magnetic resonance (CMR), it became possible to obtain a time-resolved three-dimensional acquisition of the blood flow via 4D Flow CMR. This sequence has provided a refreshed view on the estimation of the pressure drop, either peak or net, and on flow inefficiency markers [6, 11–13]. In fact, it has recently been found that 4D Flow CMR improves pressure drop association with AS prognostic markers such as left ventricular (LV) mass and 6-min walk test [14].

The validation of any method to estimate the net pressure drop has traditionally relied on pressure data obtained from reference clinical cardiac catheterisation. An implicit assumption when taking these reference recordings is that the pressure recovery is complete at the measurement point in the distal AAo. Previous studies established that a distance of 40 to 50 mm from the aortic valve (AV) was enough to avoid errors from the pressure recovery phenomenon [15, 16]. However, this assumption has never been formally evaluated.

### Review of key physical concepts

When flow enters into a constriction, it experiences an acceleration in space (i.e., advection), a conversion from potential to kinetic energy that defines the advective component of the pressure drop [17]. As a result, the pressure drops in order to accommodate the same amount of net flow through a smaller vascular lumen. The point of maximum constriction is called *vena contracta* (VC), and this is the point where EOA as well as the peak pressure drop (${\Delta P}_{peak}$) are measured. Downstream the constriction, the pressure recovery ($\Delta {P}_{rec}$) accounts for the reverse energetic conversion where the initially narrow ejection jet is gradually widened, developing a flow regime that occupies the entire vascular diameter, with decelerating flow causing an increase in hydraulic pressure. The point where the advective momentum has returned to a nearly laminar flow, is the point that defines the net pressure drop (${\Delta P}_{net}$), see Fig. 1.

In a steady theoretical fluid with no viscosity (i.e., no losses by friction), Bernoulli’s equation tells us that pressure reconversion will be total and ${\Delta P}_{net}$ will be zero (assuming that the vessel has the same diameter before and after the constriction, i.e., no net advective effects). Real fluids such as blood are inherently viscous and dynamic, resulting in an incomplete recovery of pressure. The actual stenotic burden is assessed by ${\Delta P}_{net}$ [18, 19], whereas ${\Delta P}_{peak}$, the advective pressure drop estimated by SB in clinical practice, provides a simplified surrogate of the true burden. Existing empirical evidence shows how ${\Delta P}_{net}$ correlates better than ${\Delta P}_{peak}$ with catheterization pressure recordings [3, 4]. It is important to note that these studies were performed with catheterised pressure measurements performed in the AAo in more than one location in order to try minimizing empirically the effect of pressure recovery.

Energy dissipation, comprising laminar and turbulent components, must be addressed when accounting for the true haemodynamic burden in AS. In fact, the turbulent component of the pressure drop dictates the ΔP_net_ magnitude in moderate and severe cases of AS as indicated in phantom steady flow scenarios [13]. A non-invasive assessment of this turbulent component, and of the true burden of an obstruction, is now feasible with the use of advanced CMR sequences that capture the fluctuations in flow in six spatial directions [13, 20].

## Methods

### Study aims

The aims of this study were: [1] to conceptualize and propose a non-invasive method to assess the pressure recovery distance (*PRecDist*) using measurements of flow momentum along a vessel from 4D Flow CMR acquisitions (*PRecDist-CMR*) and to directly validate against simultaneous invasive pressure recordings on a purposely designed phantom undergoing 8 different flow conditions [2]; to further validate *PRecDist-CMR* over 7 different valves and over a total of 28 physical phantom configurations against the distance to recovery defined by turbulence dissipation [3]; to explore the potential clinical importance of *PrecDist-CMR* measurement in a cohort (n = 32) of native bicuspid aortic valve (BAV) by investigating (a) the presence of potential errors of catheterised recordings by neglecting *PRecDist-CMR*, and (b) how the magnitude of stenosis and the ejection jet characteristics impact *PRecDist-CMR*.

### Non-invasive method to measure pressure recovery distance

#### Definition of distance to pressure recovery

A flow jet (e.g. after AS) experiences a quick increase and then a decline of flow momentum along the length of a vessel. The distance for pressure recovery based on momentum, the *PRecDist-CMR*, is defined as the distance between the point of maximum momentum (i.e. maximum spatial acceleration or advection) and the point when a plateau is reached downstream. 4D Flow CMR is an ideal modality to track these changes in momentum.

**Fig. 2:**
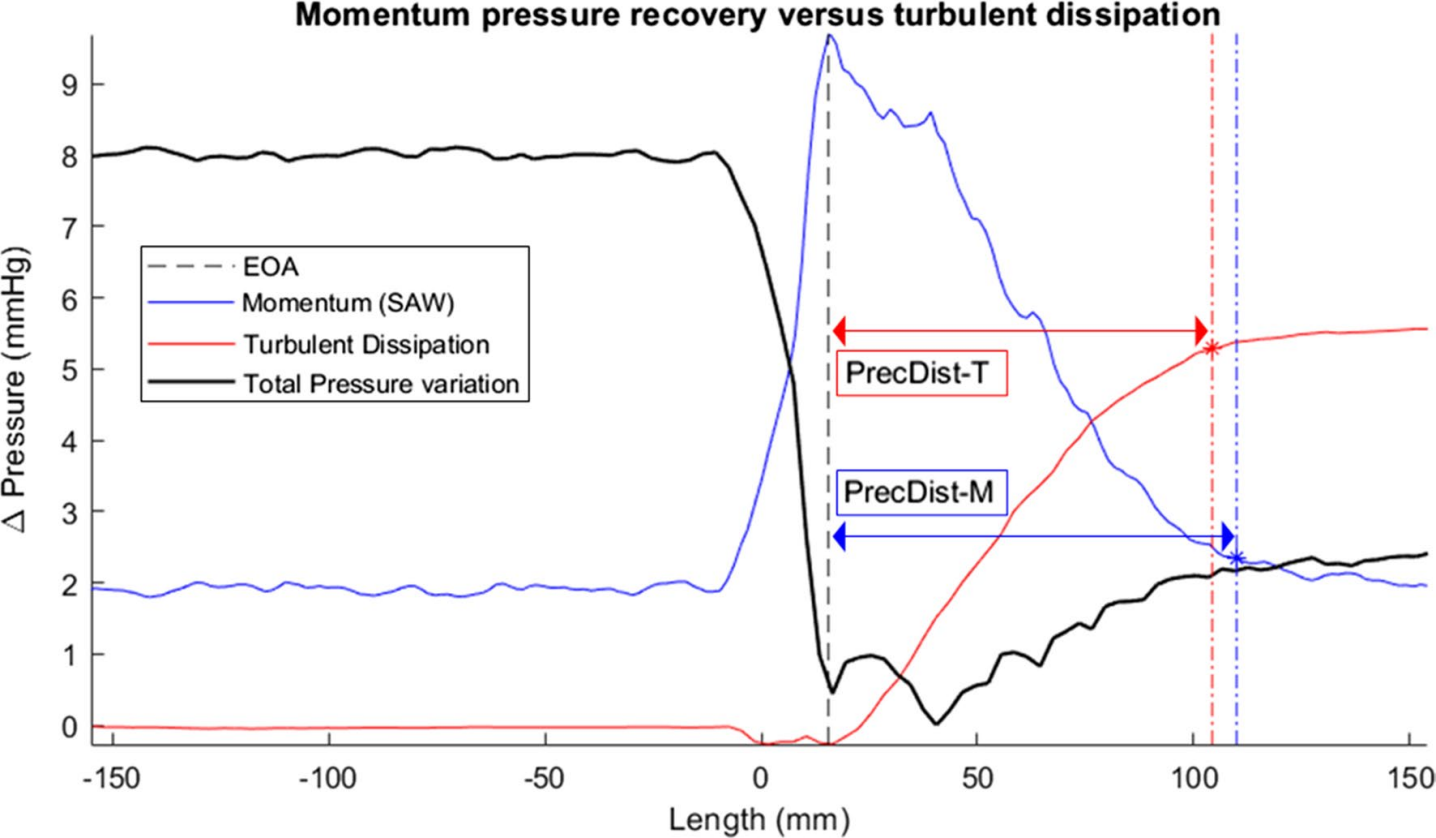
Illustration of the definition of the pressure recovery distance based on momentum (*PRecDist-M*; accessed via simplified advective work–energy relative pressure formulation—SAW) or based on turbulent dissipation (*PRecDist-T*) relative to the effective orifice area (EOA), based on an exemplary experimental result in our phantom. The distance is defined from the point of the effective orifice area (when momentum is greatest), until the recovery of 95% either the momentum created (in *PRecDist-M*) or until the accumulation of 95% of turbulent dissipation (in *PRecDist-T* )

The method formally proposes to take the EOA (cross-plane of *vena contracta* where momentum is maximum) as the starting location, and as the end location the cross-section where flow momentum has been recovered by 95% with respect to the downstream plateau (this is equivalent to say the 95% of the dynamic range of advective pressure from the peak to the baseline downstream the AV), see blue trace in Fig. 2.

Tracking turbulent dissipation, as enabled by specialised 4D Flow CMR acquisitions [21–23], is an alternative for defining and studying the pressure recovery distance. The integral of the turbulent dissipation effects along the vessel enables the estimation of ${\Delta P}_{net}$ along the length which, in turn, would match with catheterization recordings [13]. Accordingly, tracking turbulent dissipation provides the definition of the turbulent *PRecDist* (*PRecDist-T)*, see red trace in Fig. 2.

The *PRecDist-CMR* is, *a priori*, a clinically more attractive approach since it is available from any conventional flow sequences without the need for mapping turbulent flow behaviour. Thus, *PRecDist-CMR* is the focus of this study.

#### 4D flow CMR acquisition

*PRecDist-CMR* computation requires the blood velocity vector field over a region of interest comprising the stenotic region and the distal vessel considered. Therefore, any conventional 4D Flow CMR sequence can be used to quantify the blood momentum along the vessel. The acquisition parameters in this study follow the current 4D Flow CMR consensus [11]. Typical 4D Flow CMR acquisition parameters were echo time of 2.5 ms, repetition time of 5.1 ms, flip angle 7°, voxel size 1.5 * 1.5 * 2 mm, temporal resolution 40 ms and velocity encoding varying from 1.5 to 4.5 m/s in order to avoid aliasing. In-vivo acquisitions were free-breathing with electrocardiographic and respiratory gated [24].

*PRecDist-T* requires tracking the turbulent energy dissipation, and two alternatives are possible: computer flow dynamics (CFD) as performed in workbench 1 (following the description in Additional file 1), or state-of-the-art CMR flow acquisitions that maps the full Reynolds stress tensor (RSS). Two acquisition implementations that allow such acquisition are the 6D icosahedral flow encoding (ICOSA6) sequence [21–23] and the multipoint 5D flow [25]. In this study, we use the ICOSA6 sequence in the phantom workbench 2, which has been previously validated against pressure sensors [13, 21]. Acquisition parameters were described in the first publication of the dataset [26].

#### Pre-processing: segmentation and skeletonization

Semi-automatic segmentations of the vessels’ lumen with manual adjustments were performed based on the virtual angiogram image generated by the average velocity magnitude across all frames. In the scenarios where pulsatile flow occurs, only the frames with open valve were considered.

Automatic skeletonization was performed using an in-house Matlab code (The Math Works, Inc., Natick, Massachusetts, USA). As a result, the vessel centreline was generated together with a collection of cross-planes equally spaced by 1 or 2 mm, respectively in-vitro and in-vivo. Blood velocities were then interpolated at a regular grid of 1 mm within each cross-plane. Nearest neighbour interpolation was used to avoid the loss of the peak velocity events.

#### Momentum-based estimation of distance to pressure recovery: tracking advection along a vascular segment

*PRecDist-CMR* is based on the pressure difference that is needed to accelerate in space (i.e., advective pressure component of the pressure difference) the blood momentum observed at each cross-plane, and the common pressure reference is set to zero, to the null momentum. In other words, each cross-sectional plane is conceptually treated as an independent observation, measuring the pressure drop that is needed to accelerate the flow observed in the given cross-plane from an initial zero velocity configuration. Note that this is analogous to the assumption taken in the SB formulation of neglecting the proximal velocity to estimate the ${\Delta P}_{peak}$.

The accurate method to compute such advective pressure component is the simplified advective work–energy relative pressure (SAW) formulation [12]. SAW represents a physical correction of the more commonly used SB formulation: it accounts for the entire velocity profile instead of only relying on the peak velocity events [12]. Consequently, *PRecDist-CMR* was computed from the SAW pressure variation (ΔP_SAW_) along the vessel (i.e., in each cross-plane) by automatically identifying the EOA plane,- as the point of maximum SAW, and the end point as the 95% decay until the downstream baseline.

#### Turbulence-based estimation of distance to recovery: tracking dissipation along a vascular segment

The turbulent dissipation accumulated along a vessel segment defines the *PRecDist-T*. More specifically, the work–energy form of the integrated turbulent energy dissipation (calculated from velocity covariance given by the RSS) is performed along the vessel [13, 21]. Note that the covariance was masked, with the RSS negative off-diagonal terms being neglected, as recommended by Marlevi et al. [13]. The turbulent pressure loss at a given point in the centreline is thus the result of the volumetric integration of the turbulent energy dissipation from the start of the vascular domain until the cross-plane located at the given point. The distance from EOA to the 95% of the total turbulent pressure loss (over the full vessel) defines the *PRecDist-T*.

### Phantom workbench 1: validation against pressure sensors

**Fig. 3:**
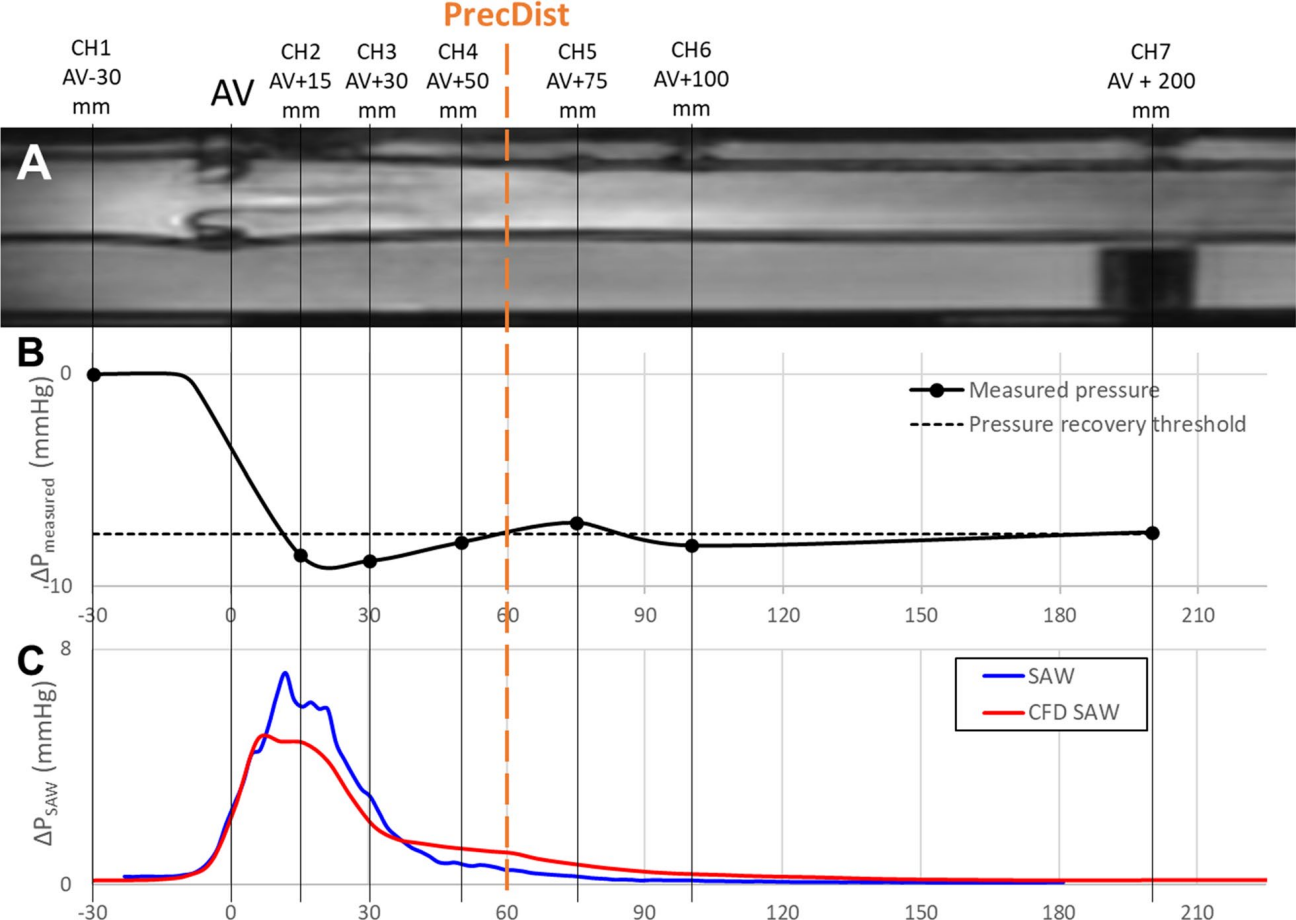
*Workbench 1*—flow phantom with 8 pressure sensors (channel 1 to 8, CH1-CH8), with their location relative to the aortic valve (AV point at X location = 0), and the respective measurement of pressure recovery distance (*PRecDist*). **A** A slice of the magnitude of the CMR image, showing most of the locations of the eight pressure ports. **B** Measurement of the pressure recovery distance from the measured pressure acquired (ΔP_measured_) and interpolated from the eight sensors. **C** Advective pressure component (ΔP_SAW_) from the 4D flow CMR data and from simulation (computed fluid dynamics, CFD) along the centerline of the phantom with 100 ml/s constant flow. Note that sensor 8 is located at 500 mm after aortic valve (AV) and is not being presented in the diagram

The validation of the proposed method against pressure measurements was based on the AV phantom developed by Gill et al. [27] where a personalized 3D-printed healthy compliant valve was implanted. This setting was imaged for 8 flow regimes (3 constant and 5 physiological pulsatile flow regimes) with peak flow rates varying from 100 to 300 ml/s, which were generated by a CMR conditional pump (CardioFlow 5000MR flow pump, Simutec, London, Ontario, Canada). The *PRecDist-CMR* was estimated and compared to the pressure recovery distance obtained from the simultaneous pressure recordings of 8 pressure ports embedded in the phantom wall (see Fig. 3 for location of the ports). Each pressure port consisted of a female Luer-lock to 1/16″ barbed port (Cole-Parmer, Vernon Hills, Illinois, USA) inserted in the wall, a 900PSI-rated Luer-lock PVC tubing (30526-14, Masterflex, Oldham, UK) attached to the port, and a calibrated and zeroed PRESS-S-000 pressure sensors connected to the tubing (PendoTech™, Princeton, New Jersey, USA).

The phantom underwent a 4D Flow CMR acquisition for each flow condition, and the respective velocity vector field was segmented. The pressure sensors were visible in the acquired images (Fig. 3A), and their location accurately determined in the CMR scanning coordinates. The points along the centreline closest to these locations were used to match those discrete locations with the CMR data. The EOA location and ground truth distance to pressure recovery from the invasive pressure measurements was obtained via the modified Akima piecewise cubic Hermite interpolation as described by Dockerill et al. (Fig. 3B) [28]—the start of the AV narrowing was considered as the knot point for the measured pressure trace. For pulsatile conditions, the instant with maximal pressure drop was taken for the computation of *PRecDist-CMR* (see Additional file 2 for further details). The agreement between the ground truth and *PRecDist-CMR* was tested for both pulsatile and constant flow conditions.

Turbulence dissipation in the cases with constant flow rate were further simulated via CFD (methodological details in Additional file 1) with the aim of an in-silico validation (Fig. 3C).

### Phantom workbench 2: validation against turbulence dissipation

**Fig. 4:**
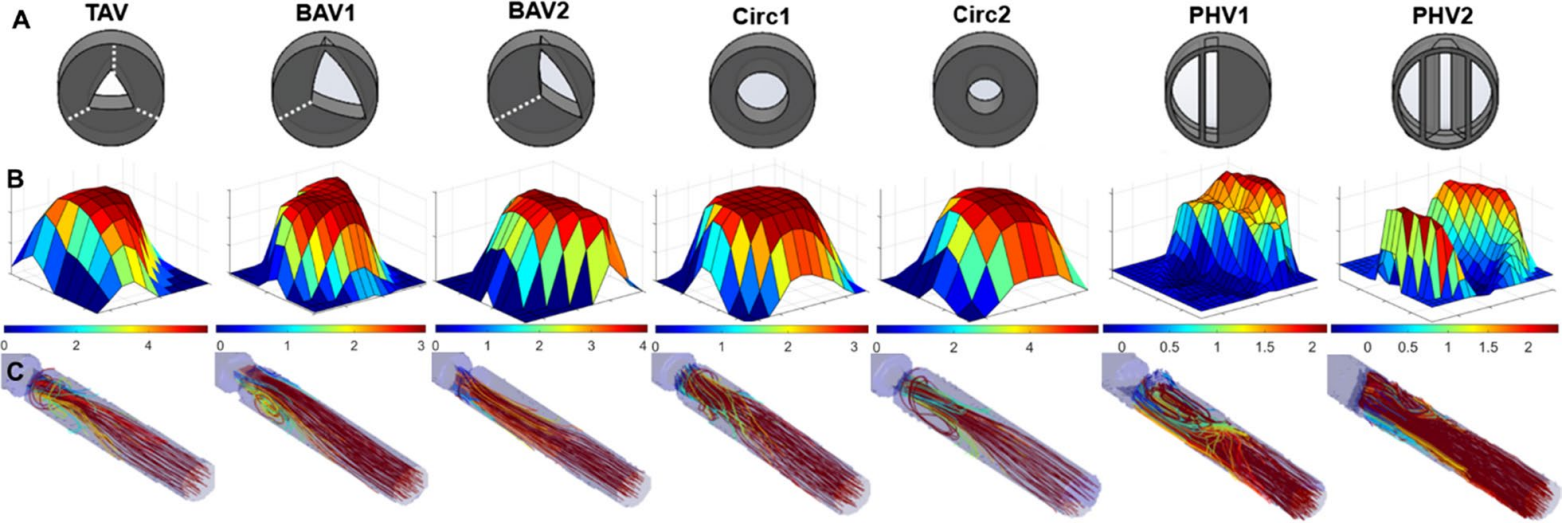
Workbench 2, a rigid pipe with constant flow and different 3D-printed heart valves to simulate aortic valve stenosis (AS). **A** The valve geometries considered—tricuspid aortic valve (TAV), two different configurations of bicuspid aortic valves (BAV1 and BAV2), two circular valves with different diameters (Circ 1 and Circ2), and two malfunctioning prosthetic heart valves (PHV1 and PHV2) [29]. **B** The respective cross-sectional blood flow profile at *vena contracta* (VC) cross-plane. **C** The respective streamlines distal from the valve

A rigid flow phantom was prepared to interexchange seven different 3D-printed AV configurations (see Fig. 4) and was consecutively scanned with 4D Flow CMR with the ICOSA6 sequence. Four different steady flow conditions with varying flow rates, were used per each valve geometry. This dataset has been used and details described in previous publications [13, 21, 29].

*PRecDist-CMR* and *PRecDist-T* were computed and the respective agreement was found. The longitudinal profiles of turbulent dissipation and advection changes were visualised and interpreted (full set provided in Additional file 3), and the ability to predict ∆P_net_ from ∆P_peak_ was tested. Ultimately, the error in the estimation of ∆P_net_ at a fixed distance of 50 mm from valve, as in the assumption of previous studies [3, 15, 16], considering the total pressure variation as the sum of momentum and turbulence dissipation.

Furthermore, a sensitivity analysis of *PRecDist-CMR* to noise and resolution was performed and is described in Additional file 4.

### Momentum recovery in subjects with bicuspid aortic valve

In a cohort of 32 BAV patients, where the BAV frequently leads to premature calcification and development of valvular stenosis, the *PRecDist-CMR* was implemented with the goal of investigating three research questions: [1] Are there errors in the ΔP_net_ catheterization measurements? [2]; Is *PRecDist-CMR* a potential surrogate of ΔP_net_? [3]; and Is *PRecDist-CMR* dependent on the flow eccentricity or vessel radius? As a preliminary exploration, the profiles of ΔP_SAW_ along the aorta were inspected to extract qualitative insights about how the pressure recovery phenomenon occurs in space.

Initially, the potential existence of errors in catheterised ground truth recordings of pressure drops was investigated by directly assessing the portion of the BAV cohort whose *PRecDist-CMR* does not occur within the length of the AAo. For the cases where *PRecDist-CMR* is longer than the AAo, catheterised recordings located at the AAo are inaccurate to capture the ΔP_net_. Besides, it is also important to avoid the end of AAo—a flow bifurcation region—with the inherent technical difficulty of catheter positioning. Thus, the actual pressure in the distal AAo was considered to be measured at ¾ of the AAo length taking into consideration previous studies that pointed out the pressure to be recovered at 50 mm of AAo [15, 16, 30]. The ratio between these cases and the total number of patients evaluated was calculated as an estimate of the prevalence of errors in ΔP_net_ by catheterization. Finally, the potential magnitude of ΔP_net_ measurement error was calculated for the patients where pressure was not recovered at ¾ of AAo length, which is the typical distal AAo point for catheterization pressure measurements (already further downstream than the 50 mm recommended).

Second, the relationship between the level of stenosis and *PRecDist-CMR* was investigated based on peak velocity (v_peak_) by comparing the patients having moderate (v_peak_ > 3 m/s) and mild (v_peak_ < 3 m/s) stenosis as stated in AS guidelines [31]. An additional analysis was done to find the correlation between *PRecDist-CMR* and the actual severity of stenosis characterised by the peak ΔP_SAW_, derived at the level of EOA.

Third, the dependency of *RrecDist-CMR* on jet eccentricity and aortic radius was investigated, given the fact that these metrics have been related to AS development [32, 33]. Large eccentricity is expected to lead to a short *PRecDist-CMR* due to the potential hit of the jet onto the wall and the sudden loss of momentum. Jet eccentricity was evaluated at two locations (the geometric orifice area (GOA) and EOA cross-sections) and by two metrics: [1] the angle between the jet and the normal of the cross-section plane and [2] the relative displacement of the jet from the center of the cross-section. The AAo radius is related to a change of impedance, due to the plunging jet penetration into the ascending AAo pool instead of within pipes of same diameter. Radius was measured at the same cross-sections (geometric orifice area (GOA) and EOA) from the virtual angiogram image reconstructed from the velocity magnitude.

### Data and statistical analysis

Reported variable values (such as pressure recovery distance, pressure drop and flow velocity) are presented as the mean ± standard deviation. When the significance of the difference between measurements was computed, a t-test was used with a significance level of p < 0.05. When testing the agreement between estimations (pressure recovery distance) and the ground truth (measured pressures or turbulent dissipation), a linear regression and a Bland-Altman plot were computed. Note that when linear regression is intended to study a potential agreement, it was enforced the zero-intercept to reflect the similarity between the variables studied, i.e. how close the linear regression coefficient is close to identity (y = x). When the goal was to search for the linear correlation between non-similar metrics, a regression was computed without enforcing the zero-intercept (such as the relation between pressure recovery distance and peak pressure drop).  Statistical analyses were performed using SPSS (version 28.0.1.1; Statistical Package for the Social Sciences, International Business Machines, Inc., Armonk, New York, USA) and Excel (v.2022; Microsoft Corporation, Redmond, Washington, USA). 

## Results

### Phantom workbench 1: validation against pressure sensors

**Fig. 5:**
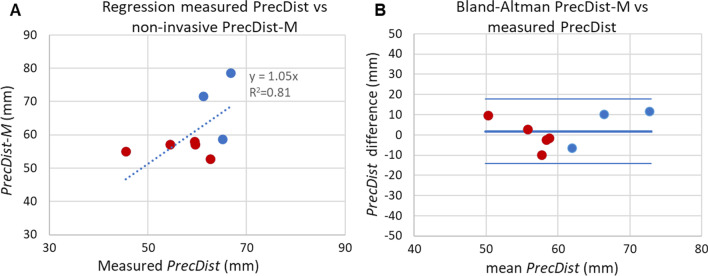
Agreement of pressure recovery distance (*PRecDist*) estimated from momentum computation (*PRecDist-M*) and measured *PRecDist* in workbench 1 including 3 constant (blue) and 5 pulsatile flow regimes (red) (total n = 8). **A** Linear regression analysis; **B** Bland–Altman plot for the agreement

Results in phantom workbench 1 revealed a good agreement (absolute error of 1.8 ± 8.2 mm, R^2^ = 0.8 and linear regression coefficient of y = 1.05x) between measured *PRecDist* (59.3 ± 6.3 mm) and the non-invasively estimated *PRecDist-CMR* (61.1 ± 8.4 mm), as shown at Fig. 5. The error made in accessing ΔP_net_ at the previously reported 50 mm from the valve was 16.7 ± 6.9%. Furthermore, in the constant flow conditions, the *PRecDist-CMR* matched the turbulent dissipation distance as measured via CFD (see Additional file 1).

### Phantom workbench 2: validation against turbulence dissipation

**Fig. 6:**
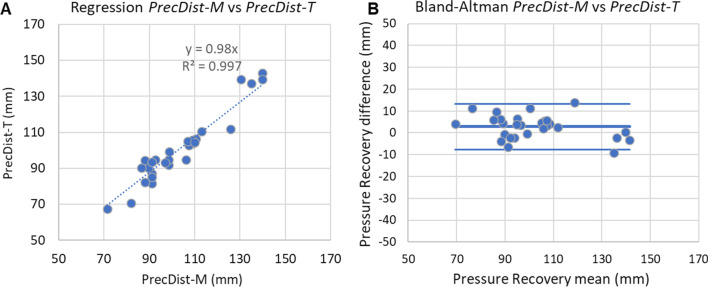
Agreement of pressure recovery distance (*PRecDist*) estimated from momentum computation (*PRecDist-M*) and turbulence-based (*PRecDist-T*) in workbench 2 including 4 constant flow regimes across 7 different valves (n = 28). **A** Linear regression analysis; **B** Bland–Altman plot for the agreement

**Fig. 7:**
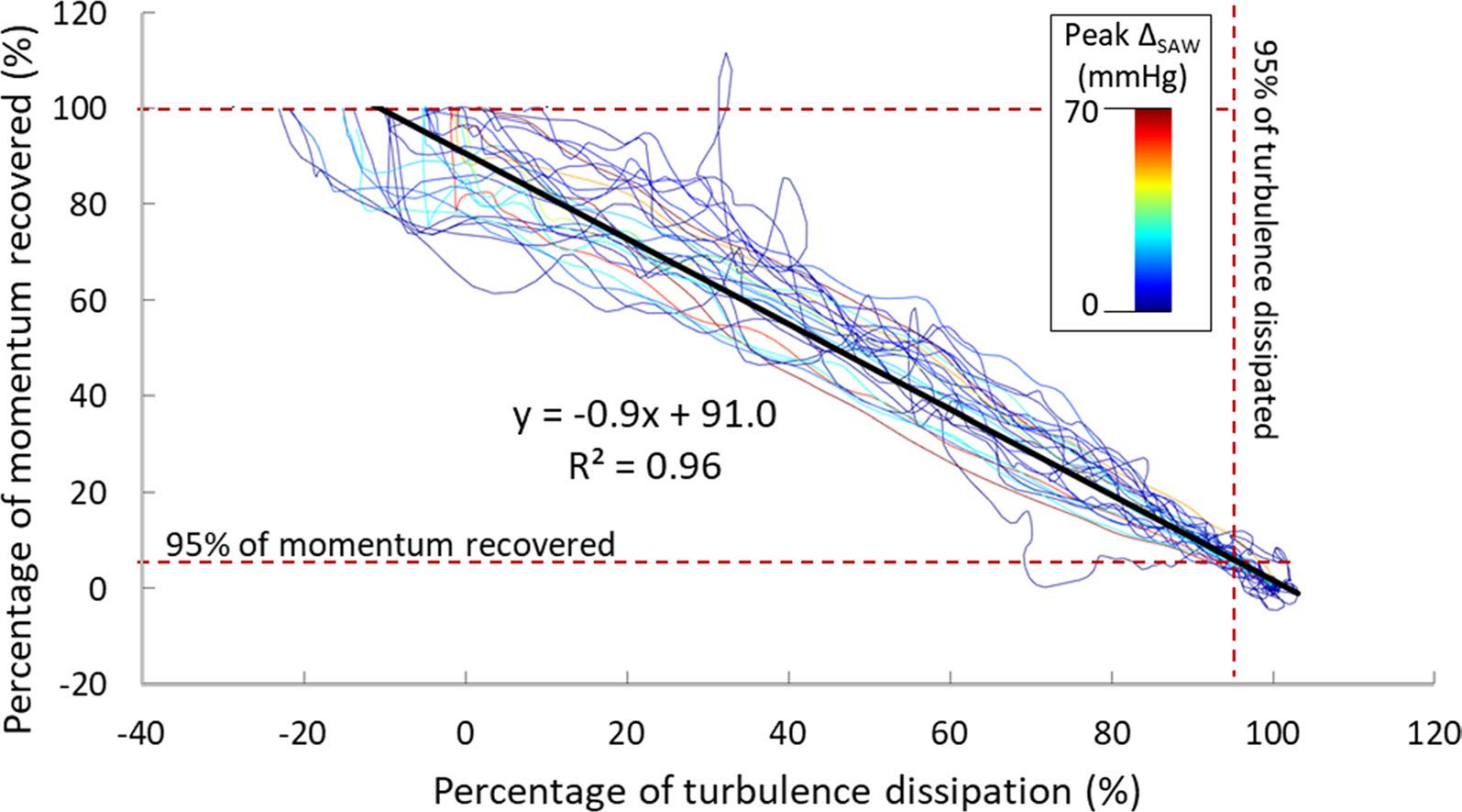
Relationship between pressure recovery (i.e., reduction of advection) and energy loss (i.e., turbulence dissipation) across the 28 experimental conditions of workbench 2, colour coded accordingly to their stenotic level (Peak advective pressure drop; ΔP_SAW_). Each line is built by the amount of advection (by ΔP_SAW_, in mmHg, Y axis) and the dissipation accumulated (by turbulence, in mmHg, X axis) at each point along the centreline of the vessel phantom. The low stenotic lines (i.e. weaker momentum in the jet) correspond to the more irregular (i.e. noisy) relationships. On opposition, the larger the stenotic level, the better the agreement. Furthermore the agreement is better as more momentum has been recovered

Results in the phantom workbench 2 reported that *PRecDist-CMR* (103.5 ± 17.4 mm) matches *PRecDist-T* (100.7 ± 19.0 mm) with an absolute error of 2.8 ± 5.4 mm, R^2^ = 0.997 and linear regression of y = 0.975x (Fig. 6). The complete set of transients, as exemplified in Fig. 2 for the 28 valves considered are reported in Additional file 3. A plot of the momentum recovery vs. turbulent dissipation revealed that the agreement is not only present at the point of 95% of recovery, but generally throughout the length of the vascular phantom (R^2^ = 0.96, see Fig. 7).

#### Estimation of net pressure drop from peak pressure drop (workbench 2)

**Fig. 8:**
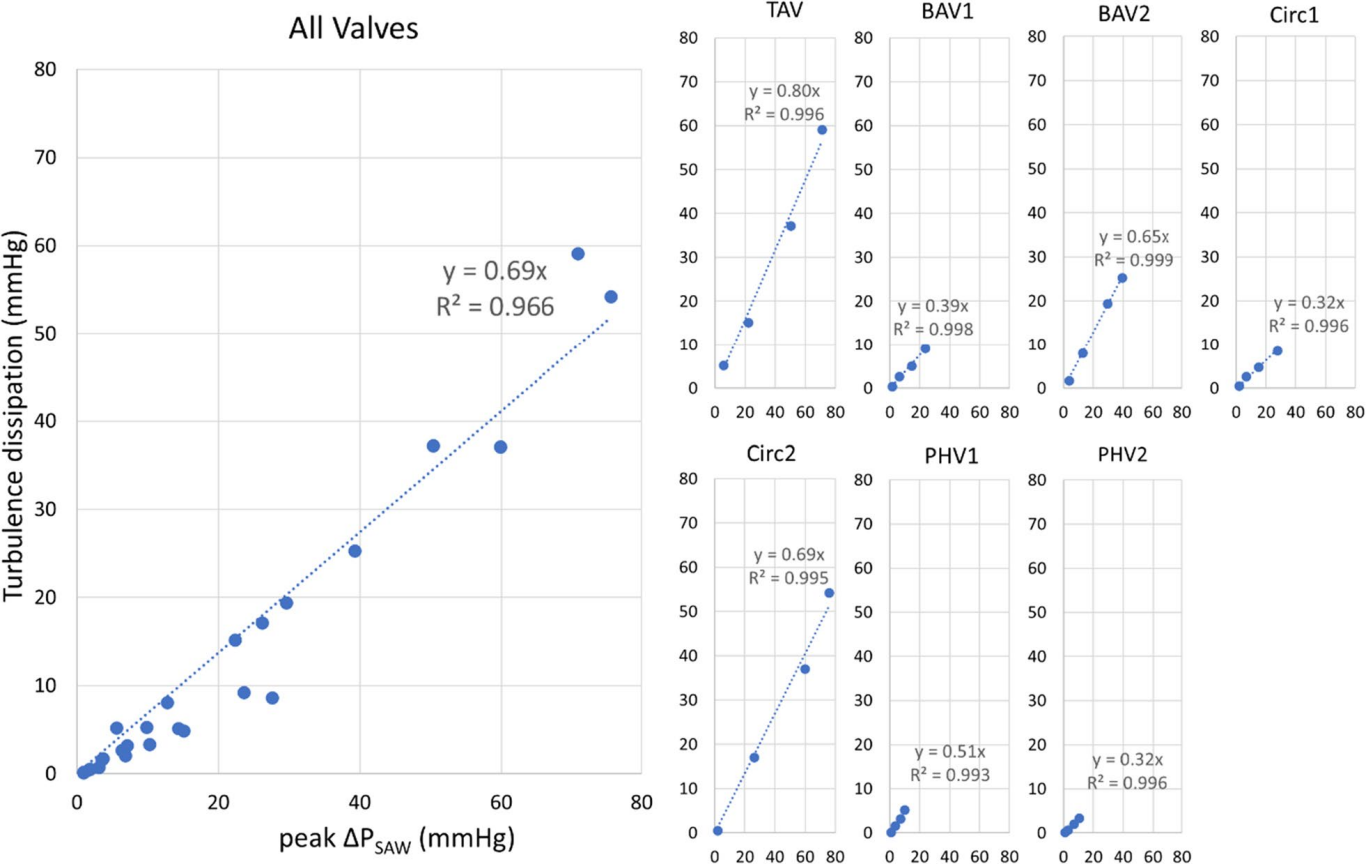
Relationship between peak and net pressure drop (ΔP), given by the comparison between the peak advective pressure drop (SAW) at the EOA and the total turbulence dissipation given by work–energy relative pressure including the turbulent dissipation component (WERP-T) for all the valves (left panel) and each valve undergoing four different flow conditions (right panels). The overall agreement (left panel) is good, but each valve (right panels) reports quite different linear regression coefficients between peak advection and net pressure drop caused by turbulent dissipation, indicating that each valve has its own peak versus net pressure drop signature. The axis of the individual valve plots has the same units as the aggregate plot

The relation between ∆P_peak_and ∆P_net_ across 7 different valves presented an excellent fit in a linear regression (R^2^ of 0.966, see Fig. 8). A more detailed inspection nevertheless revealed quite different valve-specific relationships, with linear regression coefficients ranging from 0.32 to 0.80.

The estimation of the ΔP_net_ by current clinically accepted distance to recovery (50 mm from valve) resulted in an overestimation of 42.2 ± 27.3% in ΔP_net_ in comparison with fully recovered ΔP_net_.

### Momentum recovery in subjects with bicuspid aortic valve

The *PRecDist-CMR* was 78.8 ± 34.3 mm from EOA across the BAV cohort, with 37.5% of subjects experiencing the point of pressure recovery beyond the point of the first aortic bifurcation.

Furthermore, at ¾ of AAo length landmark, 56.3% of patients still have not reach 95% of pressure recovery. For these patients, the advective pressure drop was still 24.4% ± 19.7% of the dynamic range in ΔP_SAW_—note that this is a measurement that can be assumed to be a surrogate of the error in turbulent dissipation since the relationship was approximately linear in our workbench 2, see Fig. 7.

The momentum at the EOA estimated by ΔP_SAW_ was 16.8 ± 12.6 mmHg across the BAV cohort. Subjects with clinically relevant v_peak_ > 3 m/s (n = 10; ΔP_SAW _= 31.8 ± 6.4 mmHg) typically had longer *PRecDist-CMR* (104.1 ± 25.5 mm), and in all of these subjects the pressure was not yet recovered at ¾ of the AAo length. In contrast, subjects with v_peak_ < 3 m/s (n = 22; ΔP_SAW _= 9.9 ± 7.9 mmHg) had a significantly shorter *PRecDist-CMR* (67.3 ± 31.6 mm, unpaired t-test p-value = 0.004).

**Fig. 9:**
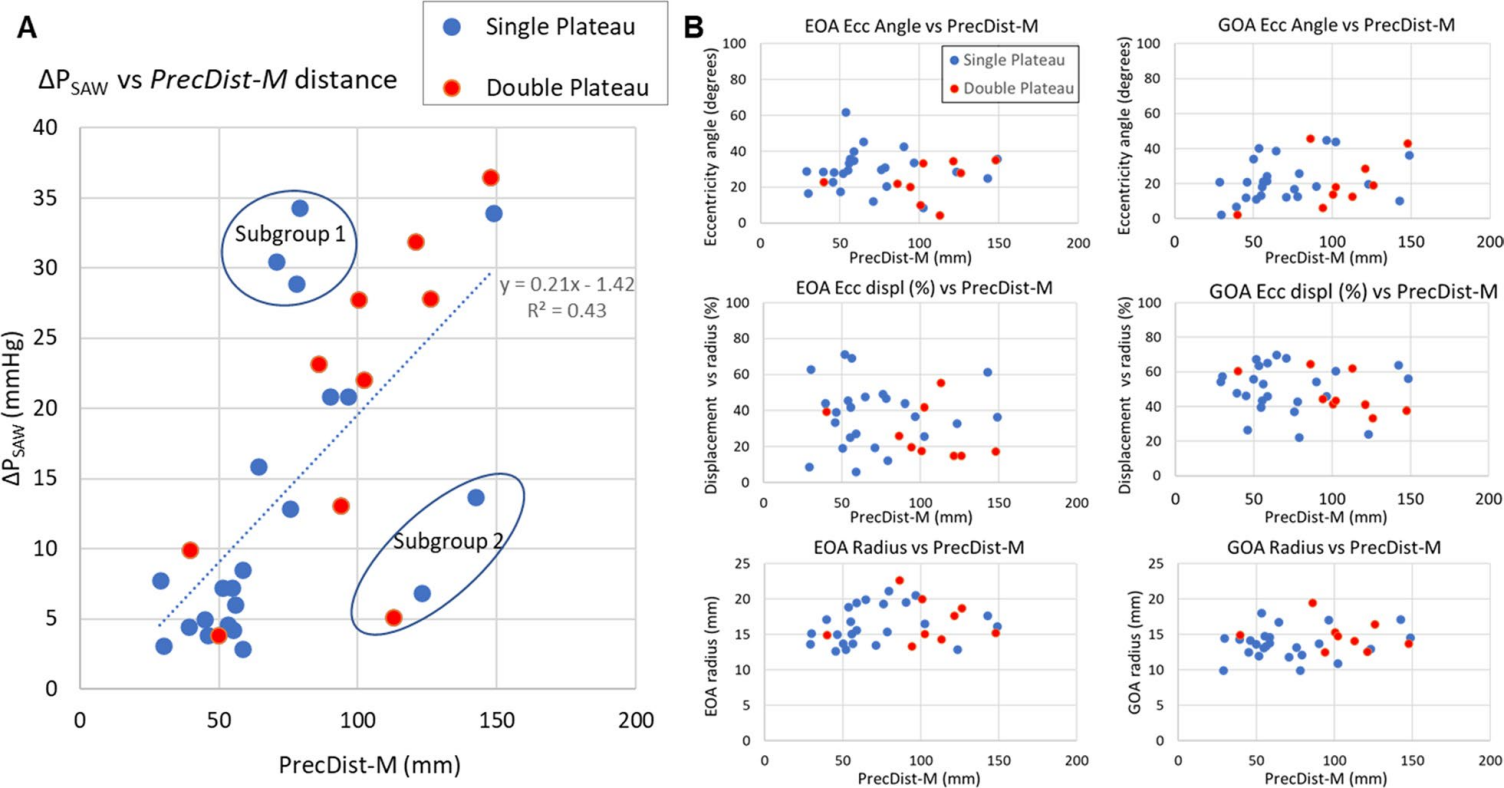
Relationship between pressure recovery distance via momentum recovery (*PRecDist-M*) and studied factors in 32 bicuspid aortic valve subjects. **A** Linear relationship with the magnitude of aortic stenotic burden (assessed by the simplified advective work energy relative pressure, ΔP_SAW_), and identification of two subgroups that do not fit well the model. **B** Lack of relationship with remaining factors considered and that have been reported to influence net pressure drop: radius, eccentricity angle (Ecc Angle) and eccentricity displacement (Ecc disp.) analysed at the effective and geometric orifice area location (respectively, EOA and GOA locations). Note that the patients presenting a single plateau of pressure recovery are presented in blue and those with double pressure recovery plateau are presented in red

The *PRecDist-CMR* has a strong relationship with the magnitude of the pressure drop (R^2^ = 0.43, Fig. 9A). *PRecDist-CMR* was nevertheless not predicted by any other metric, neither jet eccentricity or aortic radius computed at GOA and EOA locations (Fig. 9B). The diagrams of the advective momentum (ΔP_SAW_) along the centerline for the complete BAV cohort studied are presented in Additional file 5.

**Fig. 10:**
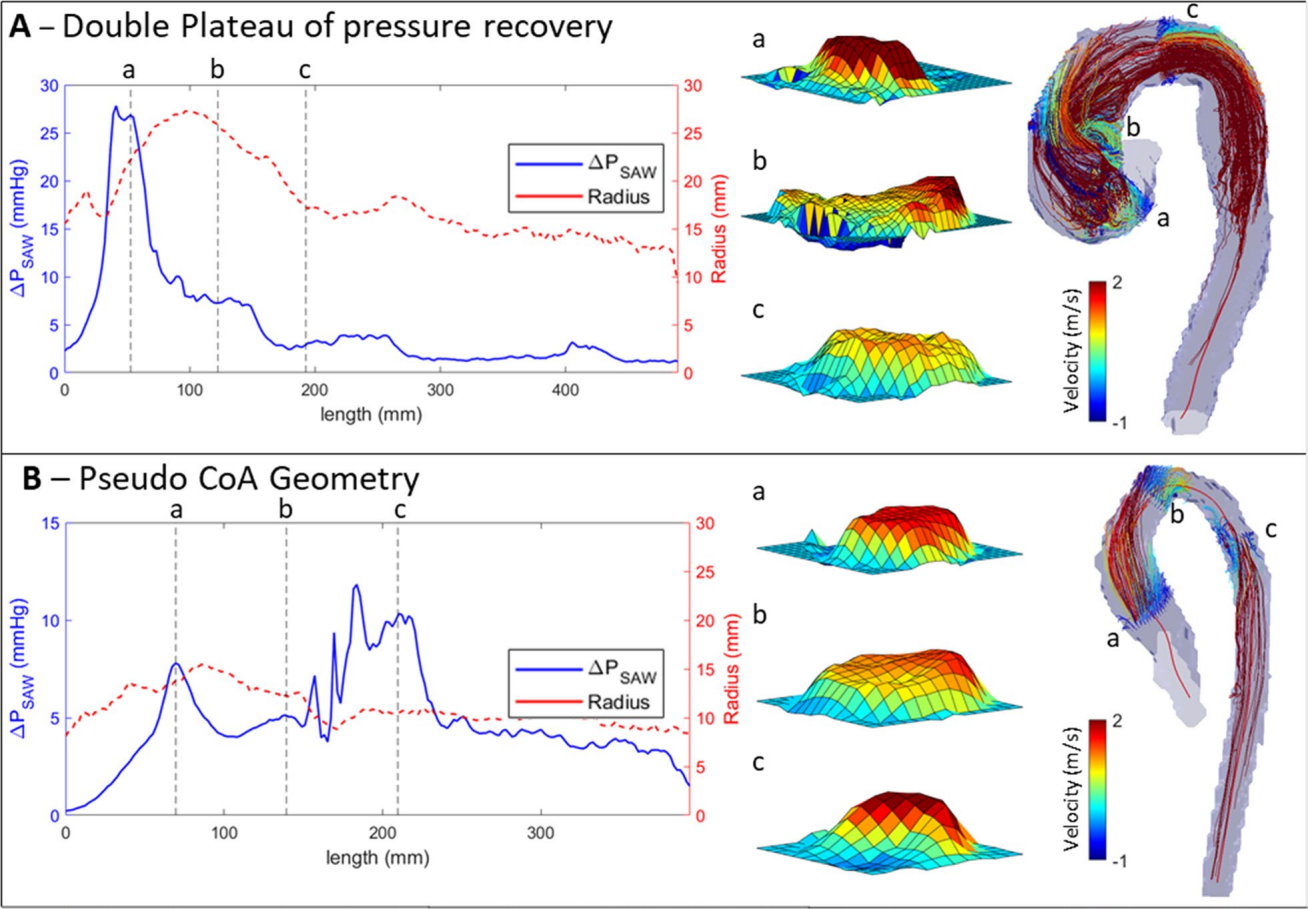
Example of a double plateau case (**A**), as well as a mild coarctation (CoA) located at plane c (**B**). Both cases display the advective momentum (ΔP_SAW_) along the centerline, the velocity profiles in 3 cross-section planes: (a) effective orifice area (EOA), (b) distal ascending aorta and (c) aortic arch; and the 3D geometry with the streamlines seeded from the same 3 cross-section planes

Some clinical cases revealed profiles of momentum recovery that were qualitatively different to those observed in the phantom workbenches. The existence of two pressure recovery plateaus was observed in 9 out of the 32 BAV cases (see example in Fig. 10A), and the second plateau was considered the right one for the computation of the distance to pressure recovery. One case presented the peak advection, not after the valve, but at a point after the arch, having an aortic geometry similar resembling an aortic coarctation (CoA)—potentially a residual mild CoA—(see Fig. 10B). In this case, the initial peak advection, the one corresponding to the valve, was the one studied.

## Discussion

The length of the pressure recovery (estimated by *PRecDist-CMR*) is significantly longer than previously reported and assumed in catheter-based validation studies: 78.8 ± 34.3 mm in 32 mild to moderate BAV patients versus 50 mm in previous works [15, 16]. Results bring experimental evidence to the assurance that once momentum is recovered, there is no further turbulent dissipation caused by the stenotic jet.

### Gold standard: from invasive catheterised recordings to flow-based estimates

Invasive catheterizations are considered the ground truth method to capture the pressure drop, but they have some limitations. Fluid filled catheters have been known to damp and distort the pressure signal [34], and only thin pressure wires should be considered to assess the true burden caused by a stenotic valve [34].

Our results demonstrate another potential source of error—the pressure recovery distance—with 37.5% of our BAV subjects not showing a complete pressure recovery (estimated by *PRecDist-CMR*) even at the end of the AAo. Looking at a conventional ¾ of AAo distance (further distally than 50 mm clinically accepted), we estimated that catheterization errors would affect 56.3% of our BAV subjects because of lack of recovery, and 100% of those with moderate AS (n = 10)—the more severe the AS, the longer the recovery distance. In addition, those patients without pressure recovered at the end of the AAo had the error in the ∆P_SAW_ estimated as 24.4% ± 19.7% versus the total pressure recovery.

This error prevalence and magnitude also challenges the existing evidence validating uni-dimensional (1D) Doppler-based assessment [15, 16]. The discrepancy found between peak pressure drops and turbulence dissipation in AS further highlights this opportunity to adopt better non-invasive estimations of the stenotic burden and improve clinical decision-making for patients [35].

An accurate estimation of the burden of AS needs to control for the location of measurement along the vessel: it must be beyond the location where most pressure was recovered from momentum. Such is fundamental for accurate AS burden quantification, non-invasively or via catheterization. Currently, there is inconsistency across intervention centers on how to robustly account for the pressure recovery when measuring the AS burden. The results here presented, in mild and moderate AS, show a longer pressure recovery distance in comparison with the clinically accepted distances. This finding indicates that novel approaches should be considered and adopted in order to better inform the AS patient diagnosis and treatment. One potential solution is to use 4D Flow CMR that captures the entire haemodynamic field and thus either identifies the location of momentum recovery as described in this work, or enables the full inspection of the aorta (until the end of the descending segment) as in the study of turbulent dissipation in AS [35]. Echocardiographic windows in the aorta could also be used to identify the location of pressure recovery, but further research is needed to establish its feasibility and accuracy.

Another solution could be the consensus to set the end of AAo as the most robust point to define ${\Delta P}_{net}$ with the risk of still missing some of the recovery (37.5% of our BAV cohort will have at least a 5% error). The fundamental difficulty is the definition of this reference point to be used to characterise the extra cardiac burden caused by AS: this metric should characterise the flow inefficiencies or energy losses caused by the valve only, although these can be generated also along the aortic arch and descending aorta [17]. Further research, and consensus, is needed to anatomically define the pressure drop that measures the haemodynamic burden caused by AS.

### Distance to pressure recovery—how to measure it?

Simultaneous acquisitions from at least two parallel pressure wires, assuming an adequate control of variable positioning along the aortic anatomy, should provide the most accurate measurement of pressure differences and the recovery phenomena. Although the theoretical ideal, achieving this clinically may not be practical [36]. In this work the *PRecDist-CMR* is proposed as a valid non-invasive alternative to measuring the pressure recovery distance, finding excellent agreement with pressure ports (linear regression coefficient of y = 1.05x and R^2^ = 0.81) and with direct measurement of turbulent dissipation (R^2^ of 0.94 with *PRecDist-T* combining in-vitro and in-silico settings).

Our results further suggest a linear relationship between relative momentum recovery and relative turbulence dissipation (see Fig. 7). The lesser regular relationships occur in velocity fields with lower magnitudes (and consequently pressure drops) and thus with less signal-to-noise ratio (SNR), given the same acquisition velocity encoding (VENC). Therefore, our results illustrate that turbulent dissipation is maximum where the jet expands downstream the narrowing, which is precisely where the biggest amount of kinetic energy is being reconverted into potential energy. This is a consequence of the evolution towards equipartition of fluid kinetic energy expenditure (into turbulent and potential energy) until steady state is reached under compressible turbulence scenarios [37]. Future studies should explore further this relationship, potentially in clinical setting.

Pressure recovery distance is quite variable in our results, a phenomena that is interpreted to be sensitive to different morphologies and haemodynamics, and ultimately has the potential to be a tool for enhancing its characterization. Moreover, given that the *PRecDist* estimated from the in-vitro phantom workbenches is also within the clinical cohort variability, it provides extra evidence that these in-vitro settings are plausible to study the pressure recovery.

### Distance to pressure recovery—length and factors involved

To the best of our knowledge this is the first study to systematically study the actual distance to recovery with the advantages—including the full aorta in the field of view over the cardiac cycle—provided by 4D Flow CMR in comparison to simultaneous pressure measurements or echocardiography. In previous pressure recovery studies, the focus has been on the magnitude of the pressure recovery, characterised to be clinically relevant mostly in patients with an AAo diameter < 30 mm [3, 7]. The assumption in these studies was that 40 to 50 mm was a distance long enough to include most of the pressure recovery based on early phantom experiments and pressure sensors [15, 16]. Nevertheless these studies focused on phantoms and patient populations with severe AS, where the pressure recovery phenomenon will less likely have an impact on the decision making [3]. The current understanding is that the pressure is recovered once the main outflow jet hits the aortic wall, at around 50 mm based on phantom evidence, and any further recovery is nominal [3, 15, 16, 30]. However, our results demonstrate that there is further energy reconversion from momentum into potential energy along the AAo.

The pressure recovery distance was found in our study to be independent of eccentricity or radius (both AV annulus and aortic root, measured at GOA and EOA locations, respectively), and to be associated with the stenotic burden given by ΔP_peak_ (R^2^ = 0.43). This finding is opposite to the factors that affect pressure recovery magnitude, reported to be dependent on eccentricity and radius [38]. Beyond eccentricity and radius, there are many factors that warrant more investigation, such as the material properties, valve morphology, aortic geometry, and their respective coupling. BAV cohort studied is a good prototype model to study these factors, since it provided a heterogenic set of *PRecDist* measurements that might be representative of the vast anatomical and physiological variation in AS.

### Implications for non-invasive surrogates

Decision on treatment of AS is informed by the ∆P_peak_ estimated using the 1D SB formulation (maximal velocity at the EOA). Such formulation has been reported to overestimate the actual pressure drop measured by catheterization [39], a result explained by two important factors neglected by the SB formulation: the pressure recovery [40] already discussed in this work, and the variable shape of the cross-section flow profile [12]. The second factor, accounting for the full cross-section velocity profile, can be solved by spatially resolved velocity at the EOA and the SAW formulation [12]. The measurement of actual energy dissipation by turbulent effects, through the computation of work–energy relative pressure integral including the turbulent dissipation component (WERP-T) and, ultimately, the virtual WERP-T formulations, are a valid non-invasive method to also account for the pressure recovery [13].

Further research is needed to optimise the assessment of the true burden of AS and enable its smooth clinical translation. Non-invasive estimates of the burden of AS should be revisited with new experiments controlling for the variable distance to pressure recovery. The challenge is a robust assessment of the magnitude of pressure recovery, or in other words the amount of turbulent energy dissipation, without an invasive pressure sensor and without excessive cost per investigation. The focus should then be the study of the factors that drive this phenomenon of recovery (or dissipation).

An interesting direction is to improve on the current surrogates, such as the ELI [7, 8]. Our results in controlled phantom scenarios suggest that tracking the advection of flow (how momentum is created and recovered) is a good surrogate to predict both the magnitude and distance of ∆P_rec_. Nevertheless, the turbulent energy dissipation, the actual source of flow inefficiencies that dictates the magnitude of the ∆P_net_, can lead to variable situations of recovery (i.e. different slopes relating kinetic to turbulent dissipation, see Fig. 8), demonstrating that the ELI that does not consider valve morphology is not a valid model for the experimental situations considered in our work.

### Potential new mechanistic insights of aortic function

The study of the relationship between distance (R*recDist-CMR*) and magnitude (SAW) of pressure recovery revealed the presence of two subgroups of outliers (see Fig. 9A). A large magnitude with a short distance (subgroup 1) alludes to the ejection jet hitting the AAo wall with the respective sudden momentum loss. A low magnitude and large distance (subgroup 2) describes a direct maintenance of the jet momentum following the curving AAo linked to the formation of helicoidal or vortical flow patterns. The coupling between AV and aorta on these 2 subgroups, probably dictated by the relative jet angulation to the AAo geometry, should be further studied to understand its potential implications in the recovery phenomena and the flow efficiency in general, given the wide spectrum of BAV valve and aorta morphologies [41].

A surprising finding is the existence in 9 (28%) of 32 BAV patients of two pressure recovery plateaus along the aorta. Such phenomena is interpreted as an incomplete pressure recovery on the 1st plateau, and it was associated in 7 out of the 9 cases with the presence of helicoidal patterns of flow in the AAo that is typical of BAV [24] where the kinetic momentum is maintained longer. Previous works described how the intra-cardiac vorticity in diastole helps to keep the blood flow momentum towards ejection [42–44], and our results suggest that the same occurs in the enlarged AAo: the helicoidal flow would contribute to sustained momentum downstream during early diastole. As such, this could be a small contributor to the reservoir function of the aorta (also called Windkessel function) without involving the elastic recoil of the wall.

Finally, the mild CoA case reported in Fig. 10B illustrates that the definition of *PrecDist-M* can be complex in a scenario with double narrowing of the vessel (from the LV until descending aorta). On the positive side, it points towards the potential early detection of evolving re-coarctation through the study of the advection along the aorta. The analysis reveals how the native geometry of an apparently healthy aorta can impact the function. The ability of the vessel to provide enough lumen to accommodate the flow momentum is the conduit function of the vessel, and 4D Flow CMR enables its comprehensive study as reported in the challenging small anatomies of babies [45].

### Limitations

Our study has several limitations. Pressure sensors in workbench 1, despite being used in controlled phantom setting, suffer from sources of errors characteristic of fluid-filled catheters such as dependence on calibration and transducer location for offset and timing as well as pressure wave reflections [46]. Nevertheless, these errors were minimized by consistent calibration against a high-fidelity catheter, and by a very short length of the fluid-filled catheter (transducers close to the phantom). Also, to minimize the impact of potential errors in the definition of the pressure recovery distance, the pressure recovery baseline was computed as the average of the most distal ports (see again Additional file 2 for the correct choices of ports for the baseline considering temporal acceleration effects).

In workbench 2 there was a local loss of flow at the inlet of the stenotic region, which does not affect the findings that are based on locations (VC and momentum plateau) downstream from this acquisition limitation.

No in-vivo pressure recovery distance was directly measured with catheterised sensors in this study, and as such there is a need of further evidence in human subjects. The ethical and operational constraints (use of invasive sensors through the valve with an intense protocol to control variable and precise positioning) limit the feasibility of this approach. Also, no in-vitro study with typical aortic arch geometries was performed. The choice of our straight pipe phantoms was driven by its simplicity and maximal control and reproducibility. The core of the proposed method to assess pressure recovery distance relies on the ability of 4D Flow CMR to sense velocity, and more specifically to sense the large velocities that define the momentum of the ejection jet after a stenosis—this is a requirement that should be easily met in-vivo.

The relationship between turbulence production and recovery of momentum has only been studied in detail in the 28 rigid phantom and constant flow conditions, and its behaviour in pulsatile in-vivo aortas is yet to be investigated. However, currently there are technical and availability limitations for acquiring varying velocity flow fields accounting for beat-to-beat variability. In the future, with the continuous improvement of acquisition and reconstruction methodologies of 4D Flow CMR sequences these issues are likely to be overcome. As such, the inference of the linear relationship between the two phenomena observed in Fig. 7 should be challenged and validated, and the potential error magnitude of the pressure recovery thereof assessed.

Finally, patients with severe AS were not included, so the effect of *PRecDist* on the respective catheterization measurements is unknown, but following the tendency displayed in Fig. 9A, it is estimated that severe AS cases will display even longer recovery distances that those reported in the BAV cohort of this study. Nevertheless, it is unlikely that the clinical decision making of severe AS patients could be affected by *PRecDist* measurement.

## Conclusion

A non-invasive methodology for the assessment of pressure recovery distance, only requiring the magnitude of the advection (i.e., velocity magnitude in each cross section) along the vessel, is proposed, validated in-vitro and applied to an initial patient cohort with AS. Using this approach, we showed that pressure recovery distances in AS are longer than previously reported and accepted, pointing to the need to refine current interventional practices. The assessment of the stenotic burden by catheter measurements using conventional locations in the AAo might be inaccurate, and the more severe the AS, the more likely the errors are. Ultimately, the present study provides evidence for the necessity of re-evaluating methods that characterise the haemodynamic burden caused by AS.

## Supplementary Information

**Additional file 1.** Turbulent dissipation versus SAW pressure recovery based on CFD simulations from workbench 1.

**Additional file 2.** *PrecDist* measurement definition in pulsatile conditions.

**Additional file 3.** Turbulent dissipation versus momentum recovery.

**Additional file 4.** *PrecDist-M* sensitivity to noise and resolution.

**Additional file 5.** Vessel radius and momentum—SAW—along the centreline.

## Data Availability

According to Wellcome Trust’s Policy on data, software and materials management and sharing, the data underlying this article will be available upon publication in a Figshare repository at: https://figshare.com/account/home#/projects/141323. Furthermore, the method to compute pressure recovery distance is available at: http://cmib.website/resources.

## Abbreviations

∆P_peak_: Peak pressure drop
∆P_rec: Pressure recovery
∆P_net: Net pressure drop
1D: One-dimensional
AAo: Ascending aorta
AS: Aortic valve stenosis
AV: Aortic valve
BAV: Bicuspid aortic valve
CFD: Computer flow dynamics
CMR: Cardiovascular magnetic resonance
CoA: Coarctation of the aorta
ELI: Effective loss index
EOA: Effective orifice area
GOA: Geometric orifice area
ICOSA6: 6D icosahedral flow encoding
LV: Left ventricle/left ventricular
LVOT: Left ventricular outflow tract
MRI: Magnetic resonance imaging
*PRecDist*: Pressure recovery distance
*PRecDist-CMR*: Pressure recovery distance defined using CMR measures of flow momentum
*PRecDist-T*: Pressure recovery distance defined from the turbulent dissipation
RSS: Reynolds stress tensor
SAW: Simplified advectived work–energy relative pressure
SB: Simplified Bernoulli
TAV: Tricuspid aortic valve
VC: Vena contracta
VENC: Velocity encoding
v_peak_: Peak velocity
WERP-T: Work–energy relative pressure integral including the turbulent dissipation component
