# Supplementary material for: Non-invasive cardiovascular magnetic resonance assessment of pressure recovery distance after aortic valve stenosis
Source: J Cardiovasc Magn Reson. 2023 Jan 30;25:5. doi: 10.1186/s12968-023-00914-3 (PMC9885657; doi:10.1186/s12968-023-00914-3)
Supplement: Supplementary file 1 — Additional file 1. Turbulent dissipation versus SAW pressure recovery based on CFD simulations from workbench 1. [file 12968_2023_914_MOESM1_ESM.docx]

# Additional file 1: Turbulent dissipation versus SAW pressure recovery based on CFD simulations from workbench 1

The CFD simulations on the phantom of workbench 1 were done with the Lattice-Boltzmann meshless software Xflow (Xflow v2021x; Dassault Systèmes, Paris, France) The geometry reproducing the phantom in-vitro setting included a closed cylindrical tube with length L=500mm and diameter d=16mm, and the tri-leaflet valve geometry segmented from MRI images (Mimics v21.0; Materialise, Leuven, Belgium). The inlet flow rate was set to equally to each of the constant flow rates (Q=100 m/s, 200m/s and 300m/s), the lateral surface of the tube and the valve surface were modelled as static walls (v=0 m/s), and the outlet was modelled as an open surface at atmospheric pressure (p=0 Pa). The blood analogue was modelled as a Newtonian fluid with density ρ=1060 kg/m^3^ and dynamic viscosity μ=0.0033 Pa∙s. The time step was set to t=5∙10^(-5)s to comply with the Courant–Friedrichs–Lewy condition; the lattice resolution was ∆x=1.6mm in the whole domain except for a refined region around the valve where ∆x=0.8mm was used to capture the turbulent phenomena with increased resolution. The Large Eddy Simulation (LES) algorithm was used to solve turbulence. The simulation was run for 15s and an initial transient period of 5s was observed in order to reach steady state. Velocity, static pressure, turbulence intensity and turbulent kinetic energy were averaged starting from 5s and extracted at the same different cross sections as the sensor locations of the tube upstream and downstream the valve. The *PrecDist-T* was computed adapting WERP-T (13) to 1D along the centerline of the phantom. The average distance between CFD derived PrecDist-M and PrecDist-T was 10.1±3.3mm.


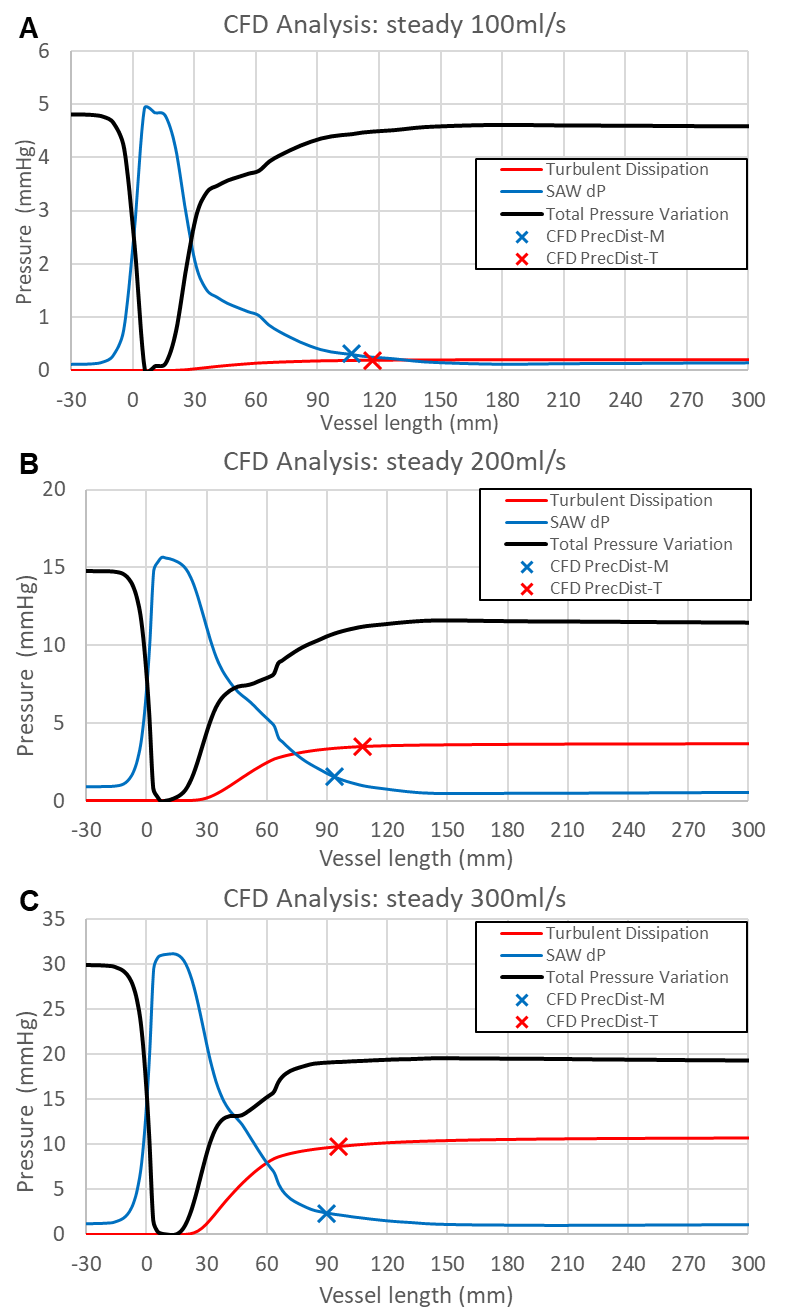


Figure S1. Computer fluid dynamics (CFD) generated profile comparison between pressure recovery distances based on momentum (PrecDist-M) and based on turbulent dissipation (PrecDist-T) for 3 steady flow conditions: A:100ml/s, B:200ml/s, C:300ml/s, as well as the total pressure variation estimation. Distance to peak turbulence matches 95% of pressure recovery.
