# Supplementary material for: Non-invasive cardiovascular magnetic resonance assessment of pressure recovery distance after aortic valve stenosis
Source: J Cardiovasc Magn Reson. 2023 Jan 30;25:5. doi: 10.1186/s12968-023-00914-3 (PMC9885657; doi:10.1186/s12968-023-00914-3)
Supplement: Supplementary file 2 — Additional file 2. PrecDist measurement definition in pulsatile conditions. [file 12968_2023_914_MOESM2_ESM.docx]

# Additional file 2: *PrecDist* measurement definition in pulsatile conditions

In pulsatile conditions, the quantification of *PrecDist-M* would require a careful choice of instant that minimises the impact of potential confounding factors. This choice is not easy, and the robust computation is done by averaging the pressure differences through the entire systolic interval as explained here.

*PrecDist-M* may be initially defined at the instant when the ΔP_peak_ is maximal across the valve, motivated by the fact that the ΔP_peak_ is fundamental in the early diagnosis of stenotic conditions (1). However, catheterised recordings used to measure the instantaneous pressures and their differences are polluted by some factors to be minimised.

The first factor that needs to be discussed is the potential impact of the temporal acceleration and deceleration of flow. This effect is illustrated by plotting the pressure difference measured at the pressure ports along the length of the vessel in 3 instances (early systole, peak velocity systole and late systole): early systole has a positive gradient (blood is being accelerated in time), and late systole has a negative gradient (blood is being decelerated in time), thus polluting the definition of the baseline (Figure S2). The instant of maximum velocity is the instant when the impact of temporal acceleration (i.e., transient component of the pressure drop) is minimal, and when the impact of spatial acceleration is maximal (i.e., advective component of the pressure drop), as described in the supplementary material of the study by Lamata et al (17). Nevertheless, the null temporal acceleration along the entire vessel will never occur - averaging over the systolic period of the cycle overcomes this issue as the acceleration has and equivalent deceleration.

Figure S2. Exemplary transients of the measured pressure drop (ΔP_measured_) depending on the systolic instance considered. Whereas in the instance of peak ΔP (corresponding to the instance when the momentum is maximum), a plateau is reached along the aorta, at the instances pre- and post-peak systolic ΔP, there is still acceleration and deceleration respectively, resulting in a reasonably constant down- and up-slope of the ΔP traces along the phantom, respectively.

The second important factor in catheter recordings is that the measurement of a single instant can be affected by pressure oscillations. These oscillations can be caused by interferences created by forward and backward pressure waves at points of impedance mismatch. In our phantom the oscillations were originated by the flow pump, that did not manage to create a rapid increase in pressure without the introduction of pressure steps (see Figure S3). Averaging over the systolic period minimises this issue as the oscillations are all integrated, cancelling their positive and negative phases.


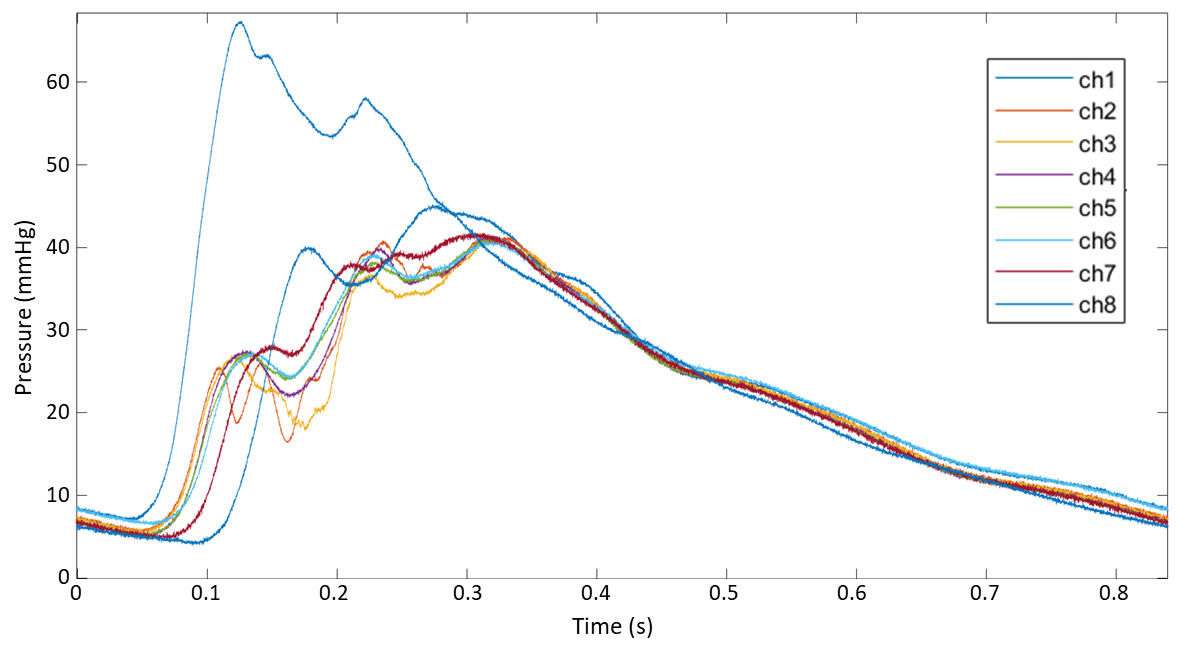


Figure S3. Exemplary transients of the measured pressure drop in the phantom workbench one, where the 8 simultaneously recorded pressures traces are shown (Channels ch1-8). The pressure oscillations are clearly visible and potentially would have a non-negligible impact in the pressure recovery distance, whereas the average trace is more robust

Therefore, the most robust manner to define *PrecDist* is to study, not the idealised instant of peak velocity, but the mean systolic pressure drop. Thus, to complement the already existing literature, we aim to stablish a further correlation between peak and mean systolic ΔP for measured pressure and PrecDist. The respective R^2^ were 0.91 and 0.64 (see Figure S4).

Furthermore, the transients of constant and mean systolic pulsatile conditions for the same peak flow rates were compared in other to visualize the potential variability on the pressure recovery profiles under pulsatile flow conditions. The magnitude of the mean systolic traces is smaller, but most importantly the transients are identical, even with the above-mentioned oscillations (see Figure S5).


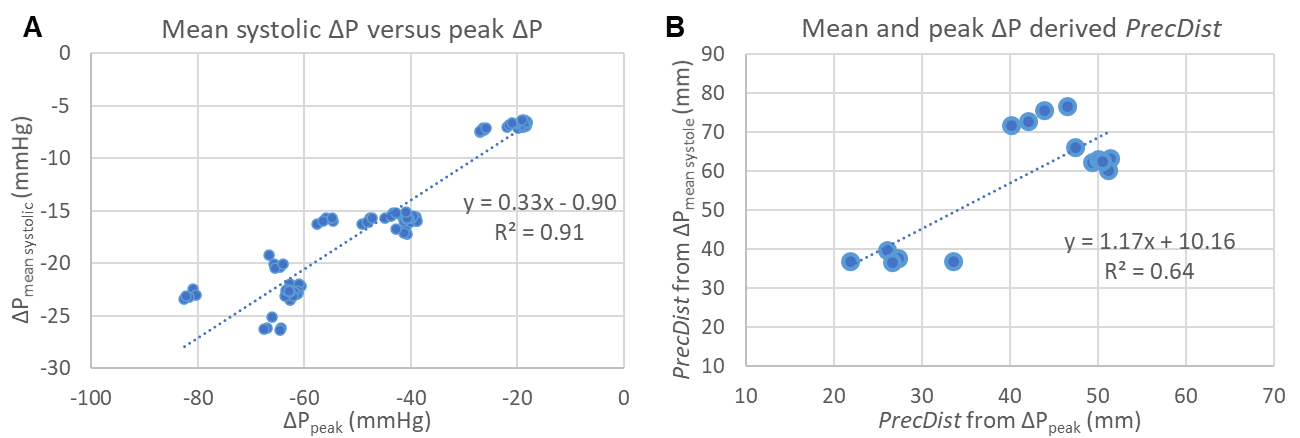


Figure S4. Scatter plots and respective correlations between (A) measured peak and mean systolic pressure drops (ΔP_mean systole_) and (B) the respective Pressure recovery distance (PrecDist).


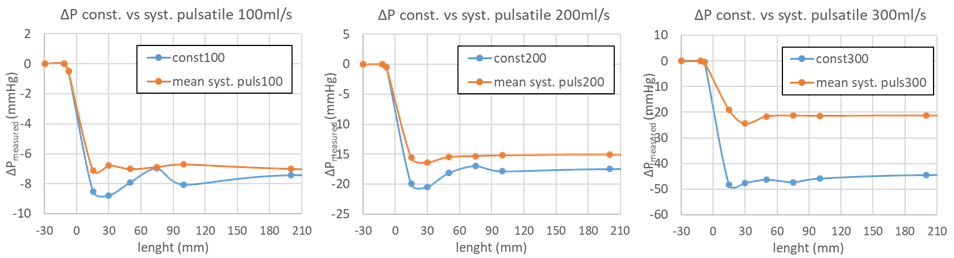


Figure S5. *Visualization of the measured pressure variation transients (ΔPmeasured) of constant (const.) and mean systolic pulsatile (mean syst. puls.) for the same trans-valvular flow rates.*
