# Supplementary material for: Non-invasive cardiovascular magnetic resonance assessment of pressure recovery distance after aortic valve stenosis
Source: J Cardiovasc Magn Reson. 2023 Jan 30;25:5. doi: 10.1186/s12968-023-00914-3 (PMC9885657; doi:10.1186/s12968-023-00914-3)
Supplement: Supplementary file 3 — Additional file 3. Turbulent dissipation versus momentum recovery. [file 12968_2023_914_MOESM3_ESM.docx]

# Additional file 3: Turbulent dissipation versus momentum recovery


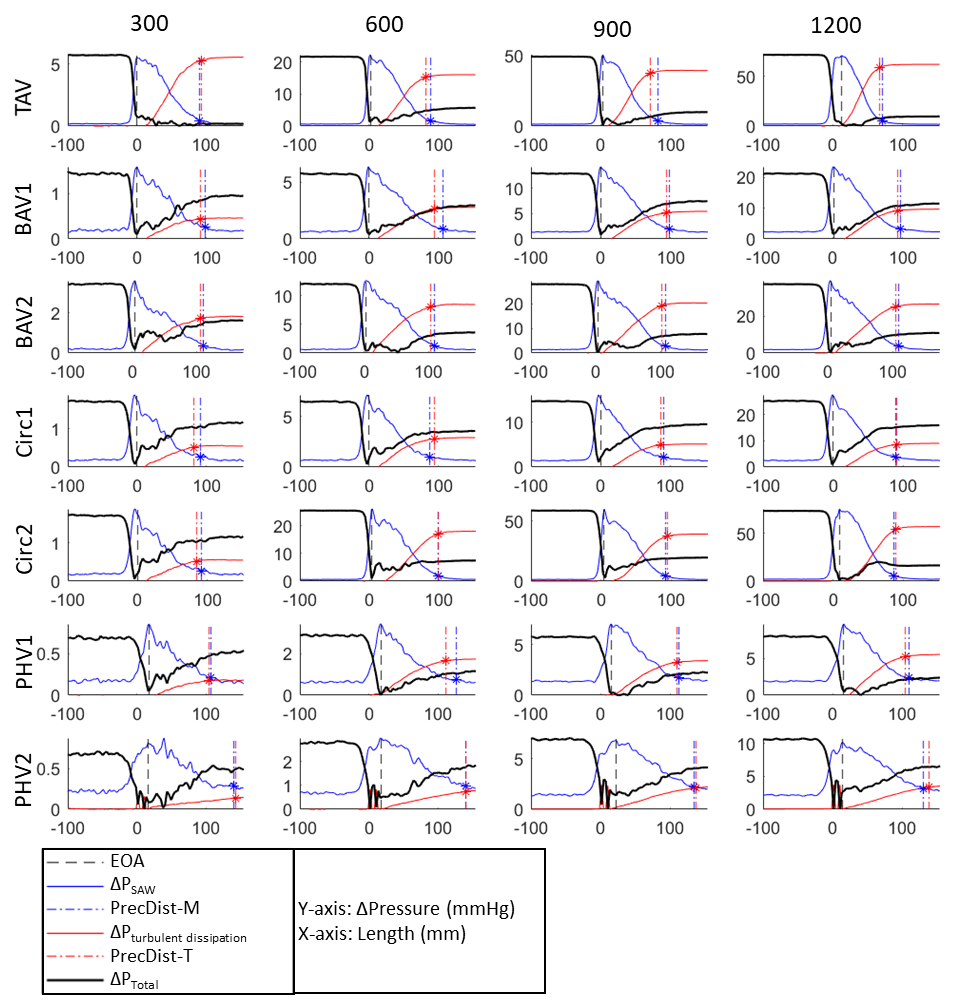


Figure S6. Profiles of changes in momentum (via simplified advective work-energy - ΔP_SAW_) and accumulation of turbulent dissipation in the phantom workbench 2 (total of n=28 datasets), showing the individual comparison between momentum and turbulence dissipation based pressure recovery distances (respectively, PrecDist-M and PrecDist-T) for each valve under each flow condition studied (flow pump revolutions per minute of 300, 600, 900 and 1200 RPM). The total pressure variation is estimated neglecting the viscous component and considering that in constant flow conditions the transient component is inexistent. Distance to peak turbulence matches 95% of pressure recovery.
