# Supplementary material for: Non-invasive cardiovascular magnetic resonance assessment of pressure recovery distance after aortic valve stenosis
Source: J Cardiovasc Magn Reson. 2023 Jan 30;25:5. doi: 10.1186/s12968-023-00914-3 (PMC9885657; doi:10.1186/s12968-023-00914-3)
Supplement: Supplementary file 4 — Additional file 4. PrecDist-M sensitivity to noise and resolution. [file 12968_2023_914_MOESM4_ESM.docx]

# Additional file 4: *PrecDist-M* sensitivity to noise and resolution

**Aim.** The sensitivity of the PrecDist-M to noise and resolution was studied for all the flow conditions of the phantom workbench 2.

**Methods.** To study the sensitivity to noise, 3 different levels of signal-to-noise ratio (SNR) were applied to the acquired velocity vector field: medium-noise (SNR = 25), high-noise (SNR = 15) and very high-noise (SNR = 5). For each flow condition of each phantom valve configuration, 5 different noise fields were created and analysed, totalizing n=140 runs per noise level. To study the sensitivity to resolution, we reduced the resolution by increasing the isovolumetric voxel size from 1.5mm to 3mm in the n=28 phantom acquisitions. The reason behind doubling the voxel space was to avoid the interference of the choice of the intra-voxel interpolation scheme. The agreement of PrecDist-M between each noise velocity field and the originally acquired was found for each noise level and plotted via Bland-Altman.

**Results.** Error magnitude is very small for permutations introduced, with nominal bias and narrow limits of agreement (see Figure S7 and S8) - the worst error has a magnitude slightly longer than one voxel. Note that the PrecDist-M agreement has a discrete profile, as the results were computed based on the original acquisition resolution (1.5mm isovolumetric).

**Conclusion**. PrecDist-M is robust to different acquisition noise levels and resolution.


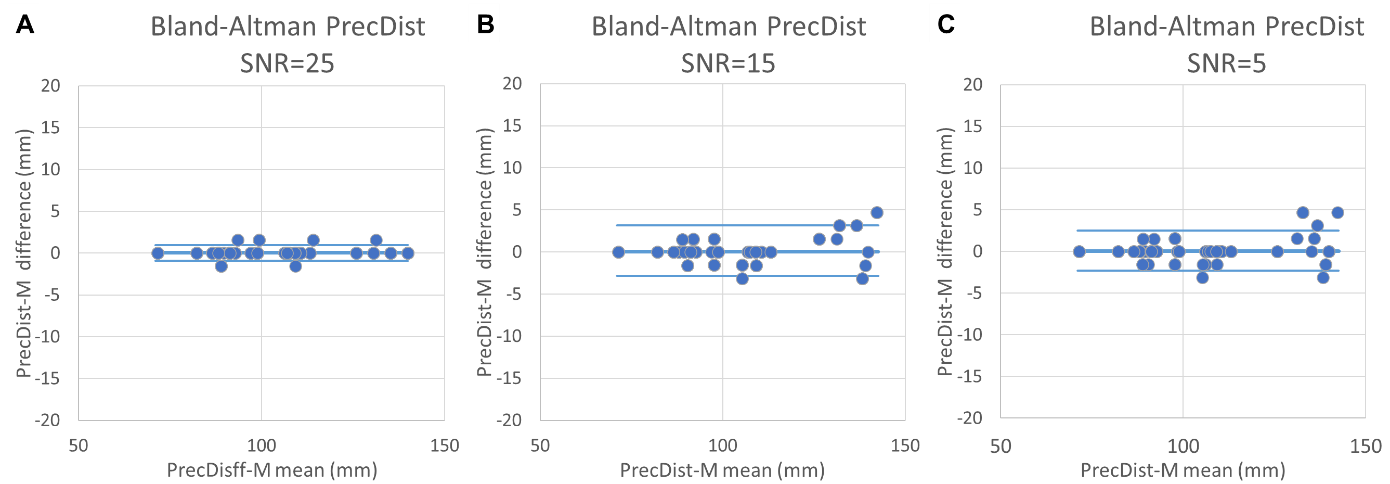


Figure S7. Agreement (via Bland-Altman plot) of Pressure recovery distance estimated from momentum computation (PrecDist-M) between original acquisition and velocity fields polluted with 3 different levels of signal-to-noise ratio (SNR): SNR = 25, 15 and 5, respectively represented in panels A, B and C (from lower to higher noise). The workbench 2 including 4 constant flow regimes across 7 different valves, was used and for each valve under each flow condition 5 noise fields were generated and analysed (total n=140 per noise level).


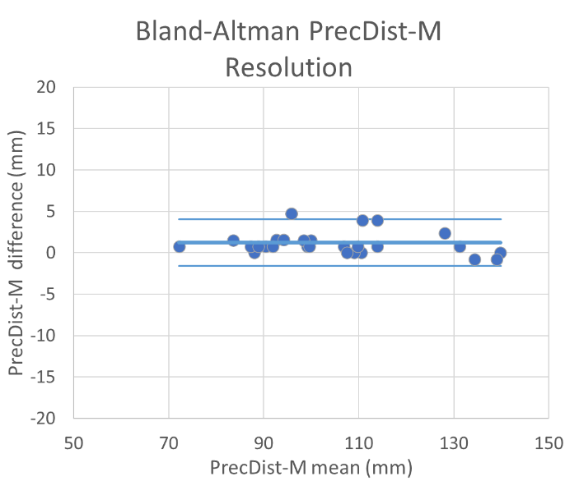


Figure S8. Agreement (via Bland-Altman plot) of pressure recovery distance estimated from momentum computation (PrecDist-M) between original isovolumetric resolution (1.5x1.5x1.5mm) versus lower resolution (3x3x3mm) tested in workbench 2 including 4 constant flow regimes across 7 different valves (n=28).
