# Supplementary material for: Non-invasive cardiovascular magnetic resonance assessment of pressure recovery distance after aortic valve stenosis
Source: J Cardiovasc Magn Reson. 2023 Jan 30;25:5. doi: 10.1186/s12968-023-00914-3 (PMC9885657; doi:10.1186/s12968-023-00914-3)
Supplement: Supplementary file 5 — Additional file 5. Vessel radius and momentum—SAW—along the centreline. [file 12968_2023_914_MOESM5_ESM.docx]

# Additional file 5: Vessel radius and momentum -SAW- along the centreline


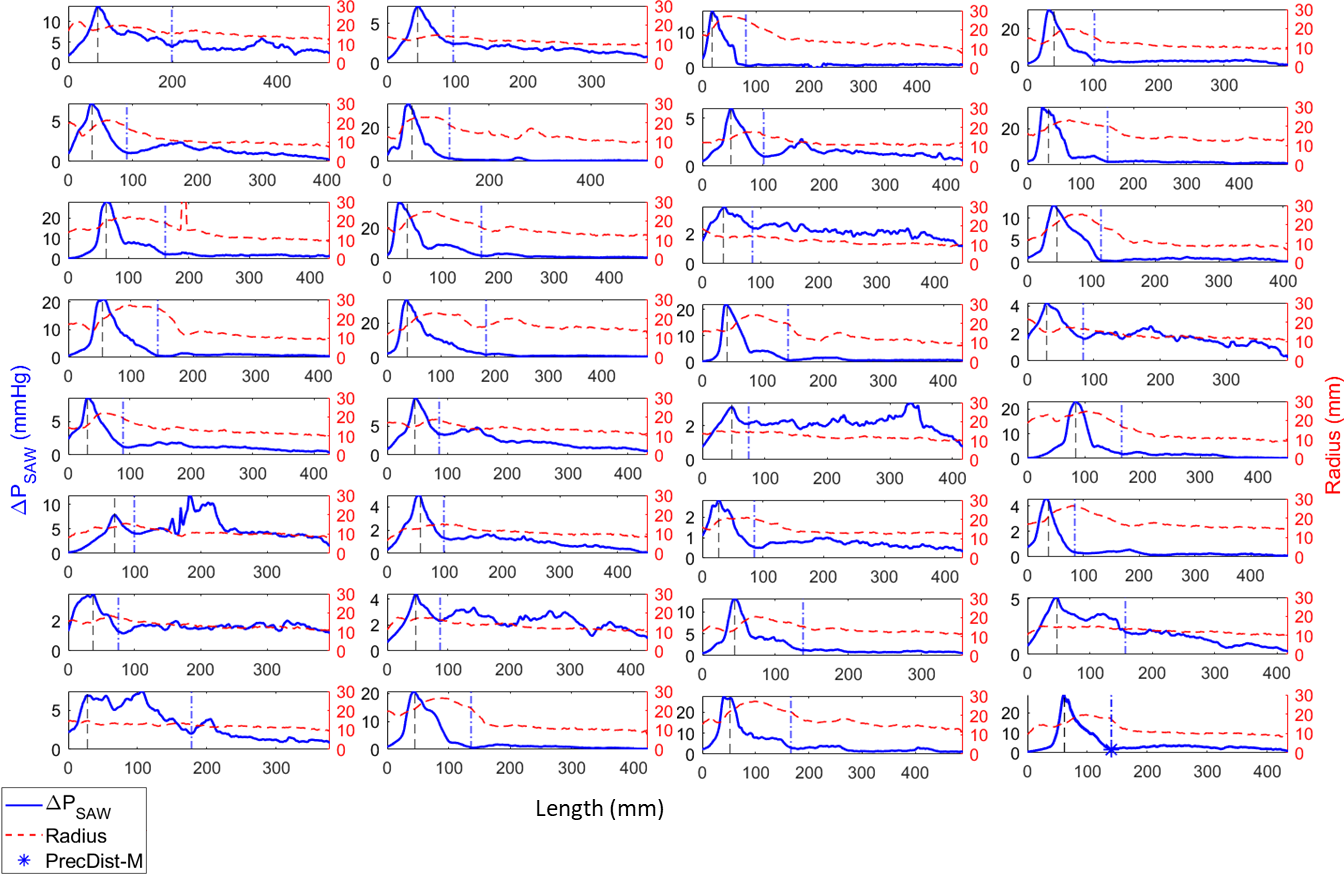


Figure S9. Profiles of momentum (via simplified advective work-energy - ΔP_SAW_), together with the respective pressure recovery distance (PrecDist-M) found, and radius along the length of the aorta for n=32 bicuspid aortic valve patients considered.
